# IL-33: targeting a muscle-to-bone signaling axis in osteosarcopenia

**DOI:** 10.1016/j.ebiom.2025.106073

**Published:** 2025-12-08

**Authors:** Reean Abdullah, Jie Chen

**Affiliations:** a Department of Cell and Developmental Biology, University of Illinois at Urbana-Champaign, Urbana, IL, USA; b Cancer Center at Illinois, University of Illinois at Urbana-Champaign, Urbana, IL, USA; c Department of Biomedical and Translational Sciences, Carle Illinois College of Medicine, University of Illinois at Urbana-Champaign, Urbana, IL, USA

Skeletal muscles and bones are intricately connected to form the musculoskeletal system that is essential for the human body's movement, stability, and support. The muscle-bone connection goes beyond the anatomical scale to manifest at the cellular and molecular levels.^1^ It is therefore not surprising that in aging, severe loss of muscle (sarcopenia) and bone (osteoporosis) are found to be concurrent in a sizable population, a condition termed osteosarcopenia (or sarco-osteopenia).^2^ Patients diagnosed with osteosarcopenia have a significantly increased risk of mortality.^3^ A recent meta-analysis of a large collection of published data has led to an estimate of the prevalence of osteosarcopenia at ∼18% among the aging population world-wide.^4^ With the world population of adults 65 years and older projected to be at 1.6 billion by 2050, osteosarcopenia presents an increasingly pressing global health problem. Understanding the pathogenic mechanisms of osteosarcopenia is necessary for the future development of effective therapeutic interventions.

In the recent issue of *eBioMedicine*, Liu et al.^5^ unveiled a novel muscle-to-bone molecular pathway that connected muscle dysfunction to bone loss, which centered on the cytokine IL-33. The work leveraged the power of human patient sample analysis and animal modeling to gain clinically relevant mechanistic insights. The authors first evaluated patients with sarcopenia, osteoporosis, or osteosarcopenia, and found significantly decreased levels of IL-33 in skeletal muscle, bone matrix, proximal bone marrow, and serum of osteosarcopenia patients. Mendelian Randomization analysis of patient data led to the identification of a cause-effect relationship between low muscle strength and low IL-33 levels, and between high IL-33 levels and low incidence of osteoporosis. The authors proposed that reduction of IL-33 in sarcopenia might lead to development of osteoporosis, and hence osteosarcopenia.

As a member of the IL-1 family, IL-33 has a double life as a nuclear factor and a secreted cytokine upon removal of its N-terminus.^6^ Through its cognate receptor ST2, the cytokine IL-33 regulates immunity and inflammation.^6^ The role of IL-33 in bone metabolism is complex, with conflicting observations reported in the literature, likely influenced by context-specific factors.^7^ To test their hypothesis, Liu et al.^5^ turned to three rat models: weight-bearing exercise (WBE), intramuscular injection of botulinum neurotoxin A (BoNT/A), which induced muscle atrophy, and ovariectomy (OVX), a model for osteoporosis. IL-33 levels increased in WBE tissues and serum, decreased in BoNT/A, and unchanged in OVX, suggesting that muscle was likely the source of IL-33. Through a series of loss- and gain-of-function experiments, the authors made a strong case for muscle regulation of bone formation via muscle-derived IL-33.^5^ The cell type(s) within the skeletal muscle which produces IL-33 remains to be determined, although fibro-adipogenic progenitors (FAPs) are a possibility.^8^

IL-33 did not affect osteogenic differentiation based on in vitro experiments with bone marrow mesenchymal stem cells (BMSCs), which prompted Liu et al.^5^ to search for potential mediators of the muscle-bone crosstalk through IL-33. Single-cell RNA sequencing performed on the epiphyseal bone marrow tissue with and without muscle-specific IL-33 knockdown, followed by differential gene expression analysis and CellChat prediction of cell–cell communications, revealed differential expression of the chemokine gene *Ccl5* in T cells.^5^ Further experiments in animal and cell models with a combination of genetic and pharmacological perturbations provided strong evidence for a signaling axis from muscle-derived IL-33 to ST2 on CD8+ T cells, which produced CCL5 to activate the receptor CCR3 on BMSCs, ultimately promoting bone formation.^5^ Therefore, reduced IL-33 production in sarcopenic muscles can result in bone loss and osteosarcopenia.

In the final stop of this tour de force of study, the authors demonstrated as a proof-of-principle of therapeutic potential that local delivery of IL-33 and CCL5 (separately) enhanced bone mass and function in the OVX rat model for osteoporosis.^5^ CCL5 has been reported to positively impact bone formation at least in mouse models.^9^ Paradoxically, CCL5, along with CCL2, CCL3, and CCL4, is found to be elevated in patients with osteoporosis.^10^ CCL5 may have context-dependent roles in bone remodeling, and redundancy among multiple chemokines in vivo^9^ should also be considered in future investigations. Although translation to therapeutics is still a long road ahead, identification of the IL-33 signaling axis contributes greatly to our mechanistic understanding of muscle-to-bone connection and presents promising new targets for future therapeutic explorations combating osteosarcopenia.

## Contributors

JC conceptualized and wrote the original draft of the manuscript. RA contributed to conceptualization, literature search and review, and editing of the manuscript. Both authors have reviewed and approved the published version of the manuscript.

## Declaration of interests

The authors declare no conflict of interest.
